# Monogamy removes constraints on reproductive tissue investment imposed by intense sexual selection

**DOI:** 10.1038/s41598-025-00180-6

**Published:** 2025-06-04

**Authors:** Abril Alexander, Maider Iglesias-Carrasco, Miguel Lozano, Francisco Garcia-Gonzalez

**Affiliations:** 1 Lancaster Environment Centre, Lancaster University, Lancaster, United Kingdom; 2 Department of Ecology and Evolution, Doñana Biological Station, CSIC, Seville, Spain; 3 GLOBE Institute, Section of Hologenomics, University of Copenhagen, Copenhagen, Denmark; 4 Centre for Evolutionary Biology, School of Biological Sciences, University of Western Australia, Crawley, WA Australia

**Keywords:** Resource allocation, Metapopulation structure, Mating system evolution, Callosobruchus maculatus, Polygamy, Episodes of sexual selection, Evolution, Experimental evolution, Sexual selection

## Abstract

A key factor influencing the reproductive success and fitness of an individual is the allocation of resources to reproductive tissue. Previous research has documented positive influences of sexual selection on the evolution of male traits such as testes size. However, few studies have focused on whether sexual selection shapes overall reproductive tissue investment patterns, and even fewer have studied it in both males and females. Furthermore, whether the ecological/demographic context modulates these effects is not known. We used experimental evolution on an insect model system to assess the independent and interactive effects of sexual selection intensity (intense [polygamy] vs. relaxed [monogamy]) and metapopulation structure (absent vs. present) on reproductive tissue investment in both sexes. Counterintuitively, we found that both males and females evolving under monogamy had higher relative reproductive tissue investment than individuals evolving under polygamy. We found no independent effect of metapopulation structure or any interactive effect with sexual selection on reproductive tissue investment of either sex. Our findings suggest that trade-offs between pre- and post-copulatory sexual selection may constrain reproductive tissue investment under intense sexual selection, and that selection under monogamy removes this constraint. Generally, our study underscores the complexities underpinning the evolution of reproductive allocation strategies.

**Supplementary Information:**

The online version contains supplementary material available at 10.1038/s41598-025-00180-6.

## Introduction

Darwin’s definition of sexual selection as selection driven by competition for mating opportunities did not account for the ubiquitousness of female multiple mating (i.e., polyandry) across animal taxa^1,2,3^. Female multiple mating extends the opportunity for sexual selection beyond copulation, potentially leading to sperm competition and cryptic female choice^4,5^. Therefore, sexual selection is better defined as the competition over limited gametes (but see^6^ for a recent redefinition of sexual selection) and, in polyandrous species, it can be divided into pre- and post-copulatory episodes of selection^4,7,8^. Sexual selection therefore selects not only for traits related to securing matings but also for traits or strategies that ensure and maximize fertilization success.

A trait closely tied to male fertilization success is reproductive tissue investment. In males, sperm competition should select for increased investment into sperm production^9,10^. Multiple studies across taxa have provided empirical evidence supporting this prediction, where increased male reproductive investment was identified in the form of larger testes and accessory glands^11-17^. Larger testes are typically associated with high sperm production^9^, whilst seminal fluids secreted by the accessory glands can enhance sperm performance or manipulate female physiology and behaviour by, for instance, stimulating female fecundity to a higher degree or reducing remating receptivity^18-22^. However, a simple but important fact is frequently overlooked when predicting relative investments by males in highly polyandrous species: mating is needed if a male is to achieve any reproductive success and it must first occur if sperm competition is ever to take place. Therefore, there is a possibility for male investment into pre-copulatory traits (e.g., sexual ornaments) that ensure high mating success to take priority over investment into post-copulatory traits (e.g., ejaculate size) when under intense sexual selection^8,23,24^.

Females have been and continue to be largely understudied in sexual selection research^25-29^, meaning that whether sexual selection intensity shapes reproductive tissue investment patterns in females is even less known than in males. Nevertheless, existing knowledge points towards possible directions for the evolution of reproductive tissue investment in females. First, male mate choice may exert selection on traits that indicate high female fecundity^30^. Second, female mate choice and female multiple mating, together with associated precopulatory and postcopulatory mechanisms of sexual selection, allow females to accrue both direct (e.g., fecundity enhancing) and indirect (e.g., good genes or compatible genes) benefits that can enhance offspring number and quality^5,31-33^. Accordingly, stronger sexual selection may promote increased female reproductive tissue investment, or else facilitate female differential allocation mechanisms, since both routes would increase the likelihood of maximizing the production of a large number of high-quality offspring. However, in cases where intense sexual conflict selects for male adaptations that are costly to females’ fecundity and survival^34-36^, females might allocate resources to resistance that could otherwise have been invested into reproductive tissue^37^.

An important aspect when assessing the consequences of sexual selection for reproductive tissue allocation relates to the ecological context. Sexual selection research has experienced a recent emphasis on the importance of eco-evolutionary dynamics in sexual selection and sexual conflict. An increasing number of studies is investigating the effects of temperature^38,39^ and other climatic factors^40^, as well as the impact of population density^41^, population subdivision^42^ or environmental complexity^43^ on sexual conflict or on trade-offs between pre- and post-copulatory sexual selection. However, no study has tested whether the allocation decisions to reproductive tissue are contingent on the way that sexual selection and ecological and demographic conditions may interact. Many natural populations are characterized by metapopulation structure, where they are subdivided into several subpopulations or demes that are connected through migration^42,44^. Such structuring may be a key aspect of ecological context that influences evolution by determining which (and how many) individuals interact and compete^45,46^. Sexual selection is expected to be less intense in smaller populations or poorly connected demes within metapopulations due to lower genetic variability, increased genetic drift and reduced interaction opportunities with mates and competitors^42,47-49^. Furthermore, metapopulation structure could potentially soften the effects of sexual selection on reproductive tissue investment, since selection within demes occurs locally, as opposed to harder selection operating in large undivided populations^50-52^.

Our study objectives are, first, to investigate the response of male reproductive tissue investment to variation in the intensity of sexual selection. Second, to shed light on reproductive tissue investment patterns in females. Third, to ascertain whether population subdivision plays a role in modulating the effects of sexual selection on reproductive tissue investment. To study whether sexual selection intensity and metapopulation structure shape reproductive tissue investment patterns, we employed experimental evolution, a powerful tool that has increasingly been used to provide insights into the evolutionary dynamics of sexual selection^53-55^. Specifically, using the seed beetle *Callosobruchus maculatus* as a model system, we set up a 2 × 2 factorial design selection experiment run for over a hundred generations to assess the independent and interactive effects of selection associated with mating system (polygamy vs. monogamy; i.e., intense vs. relaxed sexual selection, respectively) and metapopulation structure (absent vs. present) on reproductive tissue investment. If more intense sexual selection results in higher reproductive tissue investment, we should see that individuals from polygamous populations would invest more resources into reproductive tissue than individuals from populations with a history of monogamy. In contrast, when trade-offs are considered, the need for females to resist male harm and for males to secure matings under intense sexual selection could result in female resistance and male pre-copulatory trait allocations taking priority over other demands. Hence, the reproductive tissue investment of individuals from polygamous populations may be constrained. Further, due to the differences in the softness of selection associated with the presence of metapopulation structure^52^, we predicted that the subdivision of populations would soften sexual selection compared to undivided populations, and, consequently, reduce reproductive tissue investment. Therefore, we expected individuals from monogamous metapopulations, and individuals from polygamous undivided populations, to have the lowest and the highest reproductive tissue investment, respectively.

## Methods

### Experimental evolution protocol

**Fig. 1:**
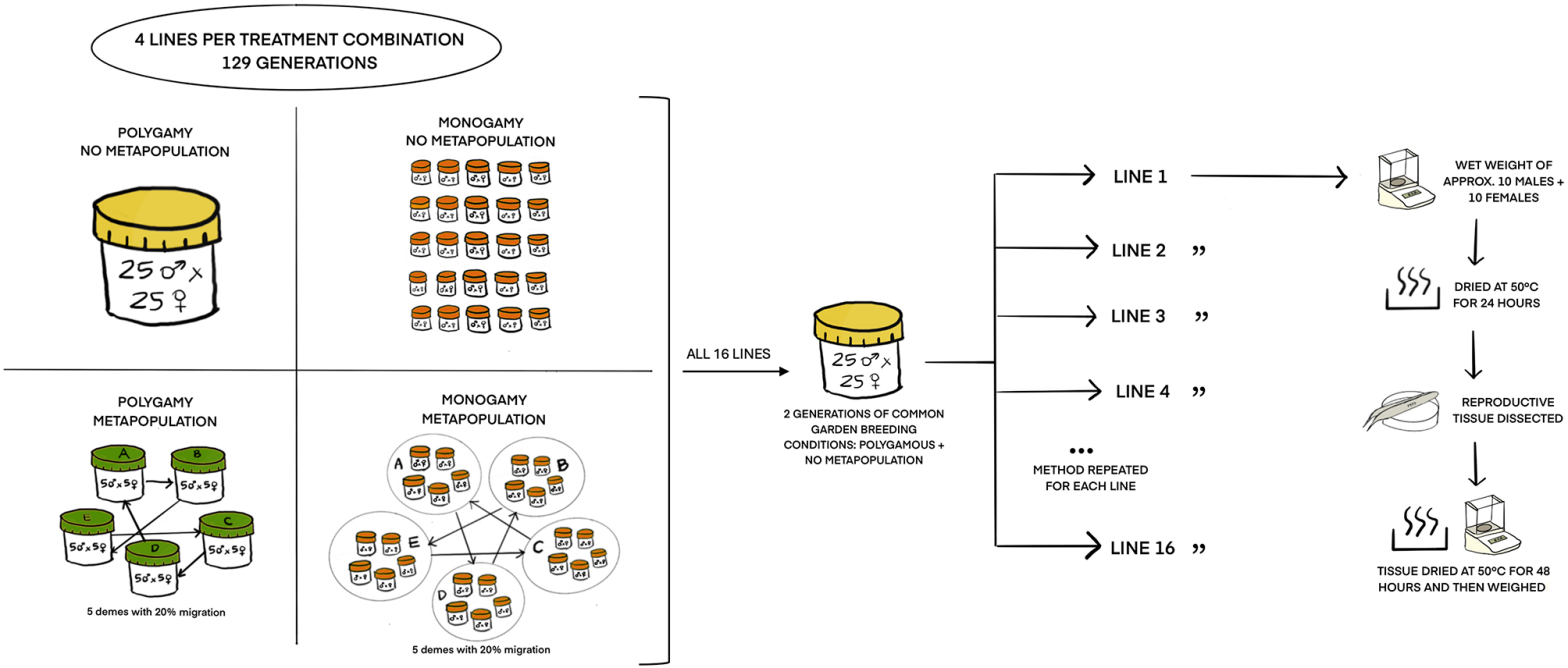
Outline of the methods, including the experimental evolution protocol and the measuring of relative reproductive tissue investment (i.e., dried reproductive tissue weight / total wet body weight) across treatment combination groups.

We used the seed beetle *Callosobruchus maculatus* (Chrysomelidae, Bruchinae) as a model system. The stock population from which selection lines were drawn was established with over 450 founding individuals from an outbred population (South Indian population) that presents sizeable phenotypic and genetic variance (see for example^42^). Further details on their establishment and maintenance can be found in the ‘Stock Population’ section of the Supplementary Materials, and full details of the selection experiment are provided elsewhere^42,52^. Briefly, the experimental evolution protocol consisted of two selection treatments with two levels each: mating system (polygamy or intense sexual selection vs. monogamy or relaxed sexual selection) and metapopulation structure (absent vs. present) (Fig. 1). The two selection treatments were crossed, with four replicate lines being established for each of the four treatment combinations, which defined the four selection regimes (polygamy and absence of metapopulation structure, polygamy and metapopulation structure, monogamy and absence of metapopulation structure, monogamy and metapopulation structure), resulting in a total of 16 selection lines. The eight metapopulation lines were divided into five demes. Connectivity between demes within each metapopulation line was imposed by relocating one randomly chosen individual from each sex and subpopulation to a different subpopulation (20% migration rate). Further details can be found in the ‘Propagation of the Selection Lines’ section of the Supplementary Materials.

At generation 129 of the selection experiment all 16 replicate lines underwent two generations of common garden breeding (conditions: undivided populations with polygamous breeding), to eliminate any environmental effects, including maternal/paternal effects, associated with the type of breeding imposed. Hence, any divergence in reproductive tissue investment could be attributed to genetic effects and genetic assimilation^53,55^.

Of critical importance for the tests in our study, *C. maculatus* are facultatively aphagous (i.e., do not require food or water) and the species is a capital breeder, obtaining all resources needed for survival and reproduction during the larval stage^56^. Thus, variation in resource availability between adults is minimised in this system. Further, variation in resource acquisition was minimized since the seeds were distributed randomly across treatments. Additionally, the standardization of the number of beans per female (approximately 64 beans available for 48 h per reproducing female; see the ‘Propagation of the Selection Lines’ section of the Supplementary Materials) ensured that each female had sufficient oviposition substrate and that larval competition was mostly absent.

## Experimental design

We tested whether sexual selection and metapopulation structure independently and interactively shape reproductive tissue investment. Such investment was inferred by measuring the weight of dried reproductive tissue of individuals from the different selection lines (see Fig. 1 outlining the methods). We aimed to obtain reproductive tissue weight data from 10 males and 10 females per line (i.e., 320 individuals in total). We took several steps to avoid dissection errors and biases. Firstly, 100 inoculated beans per line were isolated (i.e., 1600 inoculated beans in total, each bean in an Eppendorf tube with pinholes in the cap for airflow) on Day 11 of the second cycle of the common garden breeding (when larvae are between 9 and 11 days old). From Day 21 onwards, isolated beans were checked for adult emergence every 24 h. Importantly, we discarded any individuals that emerged on the extremes of the development time distribution (i.e., first day – day 24 – and on or after the fifth day – day 29 -) to avoid any potential bias associated with these animals not representing the average of the population, and because their atypical emergence day may have resulted from issues in development. This four-day range (day 25–28) captures the majority of adult emergences. Additionally, the number of individuals per line taken each day of emergence was equalized (see the ‘Assessment of the Evolution of Reproductive Tissue Investment’ section of the Supplementary Materials; Table S1). Finally, the measurement of reproductive tissue investment was done blind with respect to the selection line and selection regime. To this end, after emergence, each focal individual was transferred to a new Eppendorf tube and labelled, by a third person, with a random number. Specifically, the blind ID, treatment and date of emergence of each individual was recorded. The day after emergence, we measured the total wet body weight of individuals with a microbalance (Sartorius, ± 1 µg) using a highly repeatable method (see the ‘Repeatability of Weighing Methods’ section of the Supplementary Materials). The weighed beetles were then frozen and organized into four batches according to emergence date.

In preparation for the dissections, tin capsules were weighed and placed into one of five multi-well plates, recording the well ID and plate ID along with the capsule’s weight. Preliminary trials found the weighing method to be highly repeatable and the capsules’ weight to not vary significantly with time or temperature, allowing them to be weighed in batches. Randomly selected individuals from each of the four emergence date batches were dried at 50ºC 24 h before the dissections since preliminary trials found this protocol to facilitate dissections. This drying step before the dissections did not influence the final measurements since it was the dried reproductive tissue that was the targeted trait. We ensured that a similar number of individuals per emergence date were dissected (Figure S1). All dissections were carried out by the same person who, importantly, was blind to the individual ID (see the ‘Dissection Technique’ section of the Supplementary Materials for further details and Figures S2 and S3 for photographs of reproductive tissue). Each dissected reproductive system was placed into an individual capsule in one of the five multi-well plates. The male reproductive system was considered to include the aedeagus and testes as well as accessory glands and connective tissue, whilst the female reproductive tissue was considered to include the ovaries, ovarioles, oviducts, gonopore, spermatheca and connective tissue^57^.

The multi-well plates containing the sets of capsules and reproductive systems were placed in a drying oven at 50ºC for 48 h. The sets of capsules and dried reproductive systems were then weighed (Sartorius microbalance, ± 1 µg) using a highly repeatable method (see the ‘Repeatability of Weighing Methods’ section of the Supplementary Materials). Additionally, trials with the first few batches of dissections showed that there was no significant variation in weight when comparing sets of capsules and reproductive systems dried for 48 vs. 72 h, indicating that all water evaporates within the 48-hour drying period. Due to some mistakes during the data collection (e.g., measuring or annotation errors), the final sample size per line was reduced from the original 10 males and 10 females to 8–10 in males (mean ± SE = 9.81 ± 0.14 per line, *n* = 157 males in total) and 9–11 in females (mean ± SE = 10.38 ± 0.15 per line, *n* = 166 females in total) (Table S2). One of the removed individuals was an influential data point that exceeded 2sd from the mean (see the ‘Removed Data Points’ section of the Supplementary Materials). Overall, the final simple size per selection regime (combination of the sexual selection and population structure treatments) was: 83 individuals (40 males and 43 females) for the polygamy and absence of metapopulation structure regime, 78 (38 males and 40 females) for the polygamy and metapopulation structure regime, 82 (40 males and 42 females) for the monogamy and absence of metapopulation structure regime, and 80 (39 males and 41 females) for the monogamy and metapopulation structure regime.

## Statistical analyses

All statistical analyses were performed using R version 4.3.2 ^58^. Linear Mixed Models (LMMs) were fitted using the *lmer* function of the package *lme4* version 1.1.35.1 ^59^. LMMs were fitted to female and male data separately since *C. maculatus* exhibits a high degree of sexual dimorphism and several of the sexes’ life-history traits differ significantly^52,60^. We measured two variables per individual: wet body weight, as a proxy for body size, and reproductive tissue investment, measured as dry weight of the reproductive tissue.

We aimed to attribute variation in reproductive tissue allocation to sexual selection evolutionary history (monogamy vs. polygamy) and evolutionary history concerning the absence/presence of metapopulation structure. The response variable in the model was thus relative reproductive tissue investment, measured as reproductive tissue weight divided by wet weight. Reproductive tissue investment was controlled by body weight as our focus was to analyse relative reproductive tissue investment, and because the selection regimes are known to lead to divergence in body size (Iglesias-Carrasco, Rodriguez-Exposito, Lozano and Garcia-Gonzalez, unpublished, and see the ‘Evolutionary Responses in Body Weight’ section of the Supplementary Materials; Table S4; Figure S5). The model included the mating system treatment (two levels), and the metapopulation structure treatment (two levels), as fixed factors, as well as their interaction. Line ID was included as a random effect.

In addition to analysing the response variable as a ratio between reproductive tissue weight and whole-body weight, we ran alternative confirmatory LMMs where we analysed the response in absolute reproductive tissue weight controlled for body size by including body weight as a covariate. This model used the same fixed and random factors described above, and random slopes were also included to allow for variation in the effect of body weight on reproductive tissue weight across evolutionary lines. We present here the results of the model focusing on relative investment measured as a ratio of reproductive tissue weight / body weight because it is a simpler model that performed well in goodness-of-fit, and because the results of both analytical approaches were highly congruent. The results of the model including body weight as a covariate can be found in the ‘Reproductive Tissue Model with Body Weight as a Covariate’ section of the Supplementary Material (Table S3; Figure S4).

The significance of the fixed effects in the LMMs was assessed using Type II Wald Chi-square tests (none of the interactions in the models proved to be significant). The package ‘DHARMa’ version 0.4.6 ^61^ was used for model diagnostics. We estimated effect sizes (Cohen’s *d*) from marginal means from the model, and their associated 95% CIs with bootstrapping, using the package *emmeans* version 1.10.0 ^62^. In the models, the reference level for the sexual regime was polygamy as this is the natural mating system of *C. maculatus*, and the reference level for the population subdivision treatment was the absence of metapopulation structure.

## Results

**Fig. 2:**
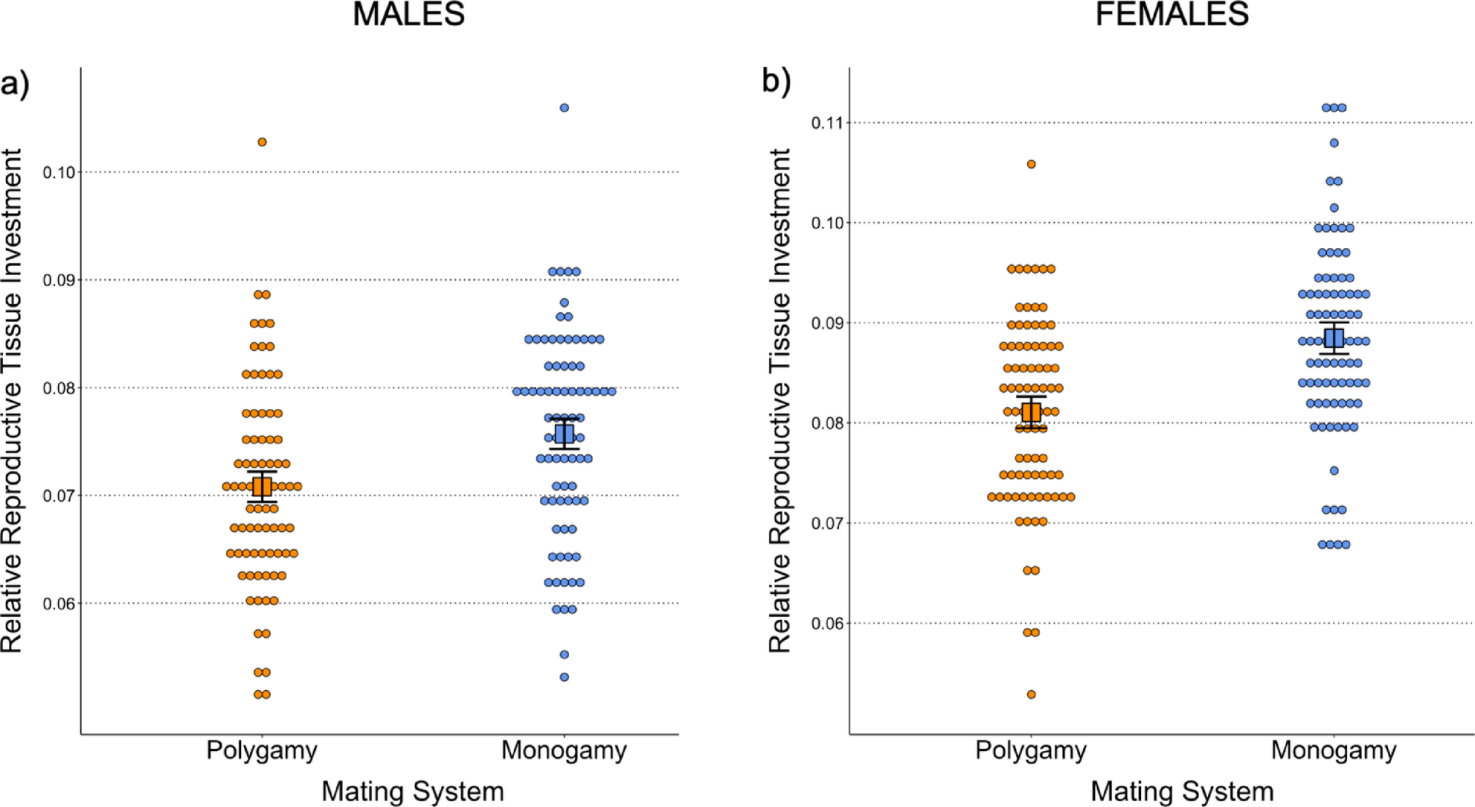
Marginal means from the models on (**a**) male and (**b**) female relative reproductive tissue investment (i.e., reproductive tissue weight divided by body weight). Each marginal mean is depicted as a square with its SE represented as error bars, whilst each group’s distribution points are plotted around its mean.

Mating system had a significant effect on male relative reproductive tissue weight (Table 1; Cohen’s *d* [95% CI] = 0.54 [0.06,1.02]), with males from monogamous lines investing 6.97% more in reproductive tissue than those from polygamous lines (Fig. 2a). Neither metapopulation structure nor the interaction between the two selection treatments were found to have a significant effect on male reproductive investment (Table 1).

Similarly, a highly significant effect of mating system on female relative reproductive tissue weight was identified (Table 1; Cohen’s *d* [95% CI] = 0.82 [0.27, 1.37]), with females from monogamous lines investing 9.39% more in reproductive tissue than those from polygamous ones (Fig. 2b). As for males, neither metapopulation structure nor the interaction between the two selection treatments explained female reproductive investment (Table 1).

**Table 1:** Effects of mating system and metapopulation structure evolutionary histories on relative reproductive tissue investment (i.e., reproductive tissue weight divided by body weight). The table shows the output of linear mixed models (LMMs) where polygamy is the reference level for the mating system treatment and the presence of metapopulation structure is the reference level for the metapopulation structure treatment. p-values significant at < 0.05 are in bold.

Fixed Effects	β	Type II Wald x^2^	Wald test df	*p*-value
*MALES*				
Intercept	0.07			
Mating System	0.01	6.10	1	**0.0135**
Metapopulation Structure	0.00	1.56	1	0.2124
Mating System : Metapopulation Structure	-0.00	0.44	1	0.5088
*FEMALES*				
Intercept	0.08			
Mating System	0.01	11.08	1	**< 0.001**
Metapopulation Structure	0.00	0.02	1	0.8915
Mating System: Metapopulation Structure	-0.00	0.74	1	0.3906

## Discussion

Interestingly, we found that intense sexual selection (i.e., polygamy) does not actually select for increased reproductive tissue investment, despite it selecting for increased body weight (see the ‘Evolutionary Responses in Body Weight’ of the Supplementary Material; Table S4; Figure S5). Instead, we found relative reproductive tissue investment to be significantly higher in populations evolving under relaxed sexual selection (i.e., enforced monogamy). It is noteworthy to have identified such variation across treatments in an aphagous capital breeder^63,64^. *C. maculatus*’ allocation of resources to reproductive investment is extremely high. To place it into context, if human investment into reproductive tissue were as high as in this seed beetle species, our reproductive systems would be substantially heavier than our heads. Estimates for human head weight as a percentage of total body weight range from 6.5 to 8.9% ^65^, whilst we found mean (± SE) reproductive tissue weight in *C. maculatus* to account for 7.32 ± 0.16% and 8.47 ± 0.23% of total wet body weight in males and females respectively (see the ‘Reproductive Investment Variation across Treatments’ section of the Supplementary Materials; Table S5). Importantly, we measured dried reproductive tissue, meaning the figures are heavily underestimating fresh reproductive tissue weight, especially since ejaculates on their own make up around 7.6% of male *C. maculatus* body weight^66,67^.

Resources are finite and must therefore be competitively allocated to different fitness components^68,69^. Hence, a possible explanation for our findings is that, under polygamy, reproductive tissue investment is constrained by trade-offs between pre- and post-copulatory sexual selection^4,7,8,23,24,70^. Importantly, in our experimental evolution, not only postcopulatory but also precopulatory sexual selection was removed in the populations in which monogamy was enforced. That is, in those populations, there is no female choice based on male traits, and males do not need to compete for access to females. Thus, since monogamous males barely have to invest in pre-copulatory traits in comparison to polygamous males, they may be unconstrained concerning reproductive tissue investment, a trait that maximizes their fitness even in the absence of male-male competition (see below). Similarly, for females, selection associated with monogamy can unconstrain reproductive tissue investment by removing the need for investment into resistance traits with which to counter sexual conflict adaptations in males^42,71-73^, and therefore enhance their reproductive success.

Sperm competition models assume trade-offs between investment into postcopulatory traits such as testes size, sperm number or sperm size, and investment into precopulatory traits that increase mating success such as mate searching, weapons or ornaments^9,24,74^. Since mating is a prerequisite for sperm competition, expenditure in mate acquisition is key for polygamous males. Trade-offs between pre- and post-copulatory traits that can constrain investment in reproductive tissue have been identified in males of various insect species, including other species of *Callosobruchus*^75-78^. Under intense sexual selection, strong last-male sperm precedence in *C. maculatus* (around 80% paternity for the last male)^79-81^ could lead to trade-offs between investment into traits that help secure matings (i.e., pre-copulatory traits) and investment into traits such as sperm numbers (i.e., post-copulatory traits)^82,83^. There are numerous examples across species of behavioural, physiological and morphological pre-copulatory traits that can provide mating advantages^1,4,84^. In seed beetles, more active males have higher mating success in *C. chinensis*^85^, whilst studies on the male production of pheromones in *Acanthoscelides* species (a genus closely related to *Callosobruchus*)^86^, and studies on the males’ capacity to detect female pheromones in *C. maculatus*^87^, indicate that mate acquisition may be influenced by physiological traits. It is also worth noting that we found males from polygamous populations to have significantly larger body size (estimated through body weight in this study) than males from monogamous ones (Figure S5). This could indicate higher investment in pre-copulatory traits under polygamy than under monogamy, since larger males have a mating advantage relative to small males^88^. Therefore, by relaxing the selective pressures impacting on mate securing and mating order, monogamous conditions would unconstrain investment into reproductive tissue.

Generally, natural selection for high reproductive tissue investment is expected under monogamy to avoid sperm limitation and to maximize reproductive output through the stimulation of female oviposition by accessory gland products^89,90^. Though some degree of male sperm depletion occurs over successive matings^91^, sperm limitation in *C. maculatus* is unlikely^92^. This also applies to our monogamous settings, where females mated with virgin males. However, the role of seminal substances on female reproductive behaviour and oviposition is well-known in the system^20,92-94^. In addition, mating provides hydration benefits to females, extending female lifespan and fecundity, at least in some contexts^95-97^. Finally, empirical work on other systems has demonstrated that enforced monogamy can reduce resource allocation to traits important in pre-copulatory competition (see, for instance^98^. Nevertheless, further exploration is required to confirm whether our findings can be explained by trade-offs between the two selection episodes. This could involve the study of pre-copulatory traits such as mating interference, mating disruption and investment into pheromones and cuticular hydrocarbons, as well as the measurement of post-copulatory traits including sperm size and number, accessory gland investment, genital size and morphology and female resistance behaviours. In addition, future studies could explore variations in the relative investment into the different elements of the reproductive system (e.g., testes, aedeagus and accessory glands in males), providing valuable insights into specific selective pressures at play.

Importantly, our findings are not the only ones to not support the general prediction of relaxed sexual selection leading to lower investment into testes size. Multiple studies on *C. maculatus*^82^ and other systems e.g.,^99,100^ have documented no evolutionary response in male ejaculate investment (testes size and accessory gland size) in response to variation in sexual selection intensity. Additionally, studies that have identified an increase in testes size with increased sperm competition risk in *Callosobruchus*^101,102^ assume a general correlation between testes size and sperm production^82^, which is highly debated. Some argue that this assumption overlooks the potential of other testicular traits such as testicular architecture, the proportion of sperm-producing tissue, and the rate of ejaculate depletion to influence sperm production rates^103,104^. Additionally, studies on multiple insect species have identified a negative correlation between testes size and ejaculate mass or sperm number^105,106^. Our findings are in line with these other studies in the sense that they do not support the most straightforward sexual selection theory prediction (i.e., a positive correlation between sexual selection intensity and expenditure). However, importantly, our study’s novelty arises from studying overall reproductive tissue investment, in both males and females, and identifying a reversed pattern from the expected one, with higher reproductive tissue investment under monogamy rather than polygamy.

We found female reproductive tissue investment patterns to match those in males. One possible explanation for this relates to the fact that, although multiple mating can provide benefits to female seed beetles^92,95,107,108^, it comes with great costs as well. Indeed, *C. maculatus* is characterised by intense sexual conflict leading to harmful (to females) male adaptations and female counteradaptations^42,72,73,109^. Enforced monogamy removes sexual conflict and therefore results in reduced male harm, which consequently leads to a decrease in female resistance and an increase in female fitness^42^. Our findings could, therefore, be explained by enforced monogamy releasing females from the need to invest in resistance traits. By reallocating resources to reproductive tissue, females could increase their reproductive success directly, as such higher investment would translate into enhanced fecundity or increased offspring size/quality.

Contrary to our predictions, we found no independent effect of metapopulation structure on reproductive tissue investment of either sex, as well as no interactive effects of the two selection treatments on the focal trait. Our predictions were founded on theoretical expectations based on variation in the softness of selection among large undivided populations and poorly connected subpopulations (see Introduction). They were also founded on several studies that identified the potential for sexual selection and selection associated with metapopulation structuring to modulate each other. For instance, metapopulation structure has been found to reverse the patterns of sexually antagonistic coevolution found in large undivided populations^42^. In addition, sexual selection buffers the negative fitness consequences of population subdivision in animals exposed to warmer temperatures^60^. The results from these studies suggest that ecological context may be key in the evolution of some sexually selected traits, including those defining the consequences of sexual conflict, and that sexual selection may play a crucial role in responding to stressful environments. However, it is important to note that not all studied traits have been responsive to the interactive effects between sexual selection and metapopulation structure. For instance, Canal et al.^52^ found no significant interaction between mating system and metapopulation structure on activity levels of either sex, despite identifying independent effects of metapopulation structure on male activity and of mating system on female activity. Reproductive tissue investment provides another example of a trait strongly and independently affected by sexual selection intensity, regardless of the spatial context of the population where it evolves. Our findings suggest that the removal of sexual selection releases resource allocation constraints, allowing maximal expenditure on reproductive tissue, which would maximize offspring production in the absence of male-male competition and female choice. Our results also suggest that variation in the softness of selection, at least that associated with the size and levels of connectivity among demes in our study when compared to undivided populations, is not large enough to result in sizeable effects affecting reproductive tissue investment. The evolution of expenditure on reproductive tissue seems to be mainly driven by marked differences in the intensity of sexual selection (presence/absence) regardless of the nuances imposed by softer or harder selection. Our study thus suggests that the evolution of reproductive tissue investment may occur independently of population spatial structuring.

We have provided insights into the patterns of reproductive tissue investment in males and females. Our findings point towards the possibility of trade-offs between pre- and post-copulatory sexual selection constraining reproductive tissue investment under intense sexual selection. Further research is needed to paint a clearer picture of the selective pressures and constraints affecting expenditure on reproductive tissue, as well as of the role of ecological context on patterns of resource allocation to reproductive or sexually selected traits.

## Electronic supplementary material

Below is the link to the electronic supplementary material.

Supplementary Material 1

Supplementary Material 2

## Data Availability

Data Availability: Data collected for this study has been uploaded as supplementary material for review and will be uploaded to Dryad upon acceptance.

## Notes

Ethics Statement: NA.
