# Supplementary material for: Monogamy removes constraints on reproductive tissue investment imposed by intense sexual selection
Source: Sci Rep. 2025 Jun 4;15:19646. doi: 10.1038/s41598-025-00180-6 (PMC12137881; doi:10.1038/s41598-025-00180-6)
Supplement: Supplementary file 2 — Supplementary Material 2 [file 41598_2025_180_MOESM2_ESM.pdf]

Supplementary Material

Monogamy removes constraints on reproductive tissue investment imposed by intense sexual selection

Abril Alexander[1*], Maider Iglesias-Carrasco[2,3], Miguel Lozano[2], Francisco Garcia-Gonzalez[2,4]

[1]Lancaster Environment Centre, Lancaster University
[2]Department of Ecology and Evolution, Doñana Biological Station, CSIC, Spain
[3]GLOBE Institute, Section of Hologenomics, University of Copenhagen
[4]Centre for Evolutionary Biology, School of Biological Sciences, University of Western Australia, Crawley, Western Australia, Australia

*Corresponding author: abrilalexander02@gmail.com

Running title: Monogamy removes constraints on reproductive tissue investment

Index

Additional Details on Methods…………………………………………………………....2
Stock Population …………………………………………………………………………..2
Propagation of the Selection Lines ……………………………………………………...2
Assessment of the Evolution of Reproductive Tissue Investment ..……………...............3
Figure S1 …………………………………………………………………………………...3
Table S1 ……………………………………………………………………………………5
Repeatability of Weighing Methods ……………………………………………………5
Dissection Technique ……………………………………………………………………...6
Figure S2 …………………………………………………………………………………...7
Figure S3 …………………………………………………………………………………...7
Removed Data Points ………………………………………………………….............8
Table S2 ……………………………………………………………………………………8

Additional Results ………………………………………………………………………8
Reproductive Tissue Model with Body Weight as a Covariate ……………………………..8
Table S3 …………………………………………………………………………………...10
Figure S4…………………………………………………………………………………..11
Evolutionary Responses in Body Weight ……………………………………………11
Table S4 …………………………………………………………………………………...12
Figure S5 ………………………………………………………………………………13
Reproductive Tissue Investment Variation across Treatments ……………………………13
Table S5 …………………………………………………………………………………...14

References ……………………………………………………………………………15

Additional Details on Methods

STOCK POPULATION

The beetles were sourced from an outbred population (South Indian population) that presents sizeable phenotypic and genetic variance e.g., see [1,2 and references therein]. The stock population was established in 2013 in a laboratory of the Estación Biológica de Doñana (Seville, Spain) with over 450 individuals and kept with large population sizes (in excess of 300 individuals) and non-overlapping generations, as described by Zajitschek *et al.* [3]. The beetles were kept in climate chambers at 29ºC, 40% relative humidity and a 12h light/12h dark cycle and were cultured in organic mung beans (*Vigna radiata*), hereafter referred to as beans. These culturing conditions mimic the semi-natural conditions that *C. maculatus* has adapted to (infestation of dry legume seed storages) and results in egg-to-adult development occurring in approx. 25 days.

PROPAGATION OF THE SELECTION LINES

Aside from the experimental manipulations to impose differential selection according to the allocated treatments (see below), the same general protocol was followed to propagate each of the 16 replicate lines. On Day 1 of the experimental cycle, 1–3-day-old virgin individuals were allowed mating and oviposition for 48 hours. The number of beans per female was standardized to 64 to make sure each female had sufficient oviposition substrate and therefore ensure that larval competition was mostly absent [2]. On Day 3, 48 hours after the breeding individuals were housed together, they were removed from the containers. On Day 11, 150 inoculated beans were randomly selected from each line. Across all treatments, a great majority of the inoculated beans had only one egg, indicating a lack of competition between females for oviposition substrate. Each of the randomly selected inoculated beans was isolated in an Eppendorf tube with pinholes in the cap to allow airflow and kept there until adult emergence. This method ensured the virginity of the individuals that would later be used as breeders for the next generation. Almost all individuals emerged between Day 25 and Day 28 and virgin adults were collected randomly from those emerged within the said time frame. This was done to avoid inadvertent selection on development time. 1–3-day-old virgin adults were set up as breeders for the next generation, starting the experimental cycle again (Day 1).

As described in the main text, of the 16 replicate lines, four were maintained under conditions of polygamy and absence of metapopulation structure, four under polygamy and metapopulation structure, four under monogamy and absence of metapopulation structure, and four under monogamy and metapopulation structure. Individuals sourced from the stock population were randomly allocated to one of 16 populations whilst keeping an equal sex ratio as all the populations were originated and kept with 50 breeders each (25 females and 25 males). The setup of each of the four treatments was as follows:

-Polygamy + Absence of Metapopulation Structure: Each of the four lines was made up of 25 breeding couples, which were placed in a 750 ml plastic container with approximately 1600 beans (64 per female). After the two days of breeding, all 25 breeding

couples were removed from the container. On day 11, 150 inoculated beans were isolated as described above. When the virgin adults emerged from the isolated beans, 50 breeders for the next generation (1:1 sex ratio) were randomly selected with the only restrictions being that the virgin adults were between 1 and 3 days old and had emerged between Days 25 and 28 of the experimental cycle. These conditions were maintained in all the other treatments as well.

-*Polygamy* + *Presence of Metapopulation Structure*: Each of the four lines was subdivided into five demes. Each deme was set-up by placing five breeding couples in a 140 ml plastic container with approximately 320 beans (64 beans per female) for the two-day breeding period. From 30 inoculated beans isolated from each deme (i.e., total of 150 inoculated beans per line), four breeding pairs were randomly selected for the next generation with the addition of one breeding pair from a different subpopulation (deme) of the population. This was done to impose a 20% migration rate between demes and therefore mimic metapopulation structure (subdivision and connectivity).

-*Monogamy* + *Absence of Metapopulation Structure*: The 25 breeding couples that made up each of the four lines were each placed in a separate 30 ml plastic container with approximately 64 beans (64 beans per female). After the two-day breeding period, six inoculated beans from each container were isolated and pooled together (i.e., total of 150 inoculated beans per line). 25 breeding couples for the next generation were randomly selected from the pool of virgin adults emerging from the 150 isolated beans, meaning monogamy is enforced in a population with no subdivision. Sexual selection and sexual conflict are greatly reduced in monogamy since enforced monogamy removes the opportunity for male-male competition and female choice [2,4,5].

-*Monogamy* + *Presence of Metapopulation Structure*: Just as in the treatment described previously, each of the 25 breeding couples was placed in a separate 30 ml plastic container with approximately 64 beans (64 beans per female). Once again, six inoculated beans were isolated from each container after the breeding period (i.e., total of 150 inoculated beans per line). However, the 25 containers were grouped in 5 groups (i.e., five demes – subpopulations - within the population) of five containers each. The beans isolated from each container were pooled with the others from their deme (i.e., a total of 30 inoculated beans per subpopulation). Four breeding pairs for the next generation were randomly selected from each subpopulation pool, with the addition of one breeding pair from a different subpopulation. In this way, monogamy was imposed and metapopulation structure was mimicked.

ASSESSMENT OF THE EVOLUTION OF REPRODUCTIVE TISSUE INVESTMENT

To avoid any bias associated with development time, the number of individuals per line taken each day of emergence was equalized: all individuals that emerged on Day 25 were selected (i.e., between 2 and 14 males, and between 1 and 13 females, across all 16 lines); 13 per sex per line of those emerged on Day 26 were randomly selected; and 8

per sex per line of those emerged on Day 27 and Day 28 were randomly selected. Each of these individuals (which were strictly 1-3 days old) was given a blind ID and kept for later measurement of reproductive tissue weight in one of four bags according to their emergence date. Individuals to be dissected were selected at random from each of the four emergence date bags. A rotation order was established (Figure S1) to balance out the number of individuals dissected per emergence date (Table S1).

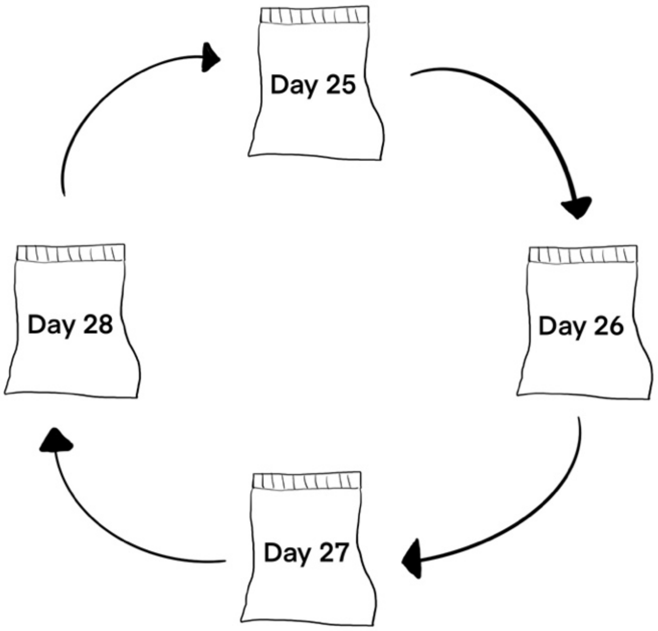

Figure S1: Diagram of rotation order between emergence date batches for selection of individuals to be dissected.

Table S1: Number of individuals dissected per emergence date in each of the 16 lines. The mean number of individuals dissected per emergence date is shown in the second-to-last row with their respective SE below them.

	Number of dissected individuals per emergence date			
**Line ID**	**Day 25 (2023-10-19)**	**Day 26 (2023-10-20)**	**Day 27 (2023-10-21)**	**Day 28 (2023-10-22)**
**1**	5	7	5	4
**2**	7	5	5	4
**3**	6	8	5	2
**4**	5	7	5	3
**5**	6	4	6	4
**6**	5	3	3	9
**7**	1	8	3	6
**8**	7	5	6	2
**9**	7	4	4	6
**10**	8	4	5	4
**11**	5	4	7	4
**12**	3	5	3	9
**13**	3	4	6	8
**14**	6	5	3	4
**15**	4	9	4	3
**16**	4	6	6	5
**Mean**	5.13	5.50	4.75	4.81
**SE**	0.46	0.45	0.32	0.56

REPEATABILITY OF WEIGHING METHODS

The repeatability of each of the weighing methods was calculated following Becker [6]:

-Wet Weight: To check the accuracy of this weighing method, its repeatability was calculated from the double measurement of the wet weight of 35 males and 35 females and was found to be highly significant between individuals of both sexes and within each sex (R across males = 0.9974; R across females = 0.9985; p in all cases = 2.20^{-16}).

-Tin Capsule Weight: The double measurement of 40 capsules' weight yielded a highly significant repeatability (R = 0.9994; p = 2.20^{-16}).

-Tin Capsule + Dried Reproductive System Weight: The repeatability of the weighing method was calculated from the double measurement of 30 capsules each containing the

dry reproductive tissue of a male and 30 capsules each containing the dry reproductive tissue of a female. The repeatability was high and significant (R across males = 0.9997, R across females = 0.9996; p in all cases = 2.20^{-16}).

DISSECTION TECHNIQUE

All dissections were carried out by the same person (AA) using the same equipment and technique. Dissections were done inside a petri dish placed under a stereo microscope using tweezers. For both males and females, the dissections began by removing the pygidium in its entirety and dousing the beetle in a saline buffer (i.e., PBS) to avoid the reproductive tissue from rupturing. The male dissections continued by gently pulling from the sclerotized portion of the aedeagus, in an attempt for the gonads and testicles to be dragged out of the beetle's body along with them. However, it was quite common for the aedeagus to break off from the rest of the reproductive system. In these cases, the dissection approach was shifted to carefully splitting and removing the abdominal cuticle until the reproductive system was fully separated from the exoskeleton. Special care was taken to not puncture the accessory glands; when this did occur, the dissection was discarded. The male reproductive system was considered to include genitalia as well as all accessory glands and connective tissue. For females, after the removal of the pygidium and the addition of the PBS, the dissections continued by removing the ventral abdominal sternites until the bursa copulatrix was exposed. To attempt to drag the reproductive system out of the female's body, the tweezers were carefully pressed against the bursa copulatrix. If this method failed, the abdominal tergites and sternites were removed in their entirety until fully separating the reproductive system from the exoskeleton. The female reproductive system was considered to include the ovaries, ovarioles, oviducts, gonopore, spermatheca and connective tissue [7]. Each dissected reproductive system was placed in an individual capsule in a multi-well plate, ensuring the tissue was covered in the smallest volume of PBS possible to minimize the effect of salts on the final weight. The tweezers and petri dish were washed and dried between each dissection to avoid contamination between individuals. Figures S2 and S3 show examples of the dissected reproductive tissues for males and females, respectively.

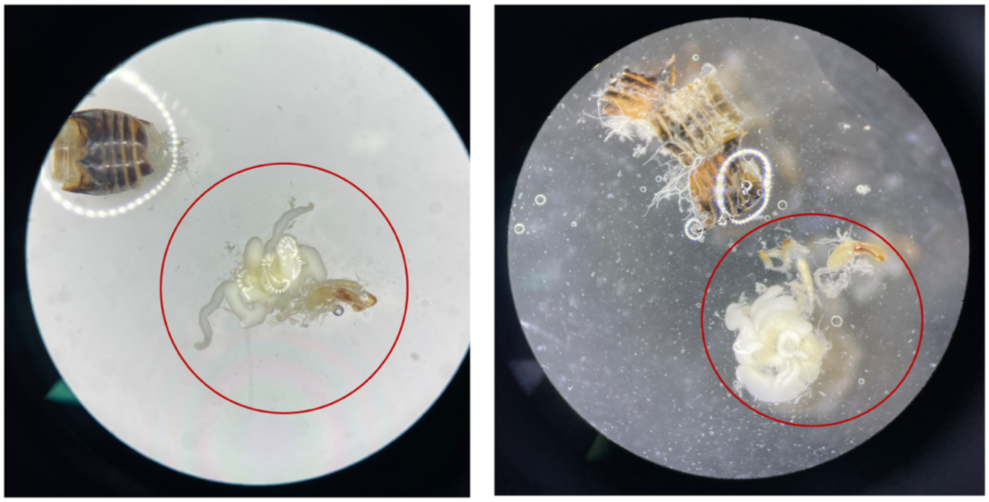

Figure S2: Photographs of dissected male reproductive tissue (circled in red) alongside abdomen exoskeleton taken through the lens of a stereo microscope.

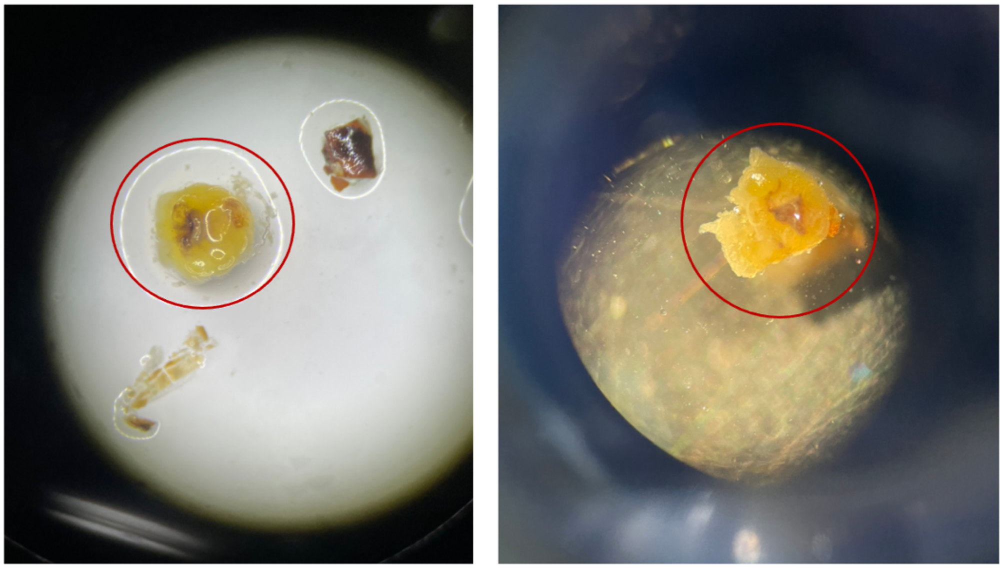

Figure S3: Photographs of dissected female reproductive tissue (circled in red) taken through the lens of a stereo microscope. Left photo also shows pieces of abdomen exoskeleton.

REMOVED DATA POINTS

Although the goal was to dissect 10 males and 10 females per line, the number of data points per line ranged from 8-10 in males (mean ± SE = 9.81 ± 0.14 per line, n = 157 males in total) and 9-11 in females (mean ± SE = 10.38 ± 0.15 per line, n = 166 females in total). This was partly due to the removal of certain dissected individuals from the dataset: three had to be removed because of errors when annotating their blind ID, one due to a clearly erroneous weight measure, and one due to its weight exceeding 2sd from the mean and also because it was an influential data point. Additionally, the blind setup of the experiment meant that someone aside from the dissector had to supervise the daily selection process for beetles to dissect to ensure that 10 individuals per sex were dissected across all lines. However, some miscommunications in that process led to some extra females being dissected in seven lines. The total number of individuals dissected per treatment and selection line is displayed in Table S2.

Table S2: Number of individuals dissected per treatment and selection line.

Treatment	Line ID	Male Count	Female Count
Polygamy No Metapopulation Structure	1	10	11
	2	10	11
	3	10	11
	4	10	10
Polygamy Metapopulation Structure	5	10	10
	6	10	10
	7	8	10
	8	10	10
Monogamy No Metapopulation Structure	9	10	11
	10	10	11
	11	10	10
	12	10	10
Monogamy Metapopulation Structure	13	10	11
	14	9	9
	15	10	10
	16	10	11

Additional Results

REPRODUCTIVE TISSUE MODEL WITH BODY WEIGHT AS COVARIATE

An alternative LMM was tested, where the response variable was reproductive tissue weight in its raw form instead of its ratio to wet weight, and wet body weight was instead included as a covariable. Random slopes were fitted to account for variation in the effect

of the covariable (i.e., wet weight) on the response variable (i.e., reproductive tissue weight) across selection lines. The fixed effects included were the same as in the model shown in the main text (Results section): the mating system treatment (two levels), the metapopulation structure treatment (two levels), and the interaction of these two factors. Line ID was included as a random effect with random intercepts. The inclusion of random intercepts and slopes was done to avoid inflation of type I error [8-10].

In the case of males, metapopulation structure and the interaction between the two selection treatments did not have a significant effect on male reproductive investment (Table S3), as was the case in the model using reproductive tissue weight divided by body weight as the response variable. However, whilst the model using the weight ratio did find a significant effect of mating system on male reproductive tissue weight (see the main text, Results section), the alternative model did not. Nevertheless, this was a marginally non-significant effect (χ^2= 3.00, P = 0.0831; Table S3), and the effect size indicates that the existence of a true effect cannot be ruled out with confidence (Cohen's *d* [95% CI: 0.42 [-0.15, 1.00]). The lack of a clear significant effect may be due to the conservative nature of random slopes models [8,9]. Additionally, the direction of change is similar in both models, with monogamous males exhibiting higher reproductive investment than polygamous males (Figure S4a). Therefore, the outcomes of the alternative male model (i.e., the model using total body weight as a covariate) were interpreted as confirmatory of the model using weight ratio. In the case of females, the alternative model's outcomes fully matched those of the model using weight ratio. Mating system was found to have a significant effect on female reproductive tissue weight (χ^2 = 10.22, p = 0.0014; Cohen's *d* [95% CI] = 0.82 [0.22, 1.42]; Table S3), with monogamous females having higher reproductive tissue investment than polygamous females, as in the simpler model (Figure S4b). Additionally, metapopulation structure and the interaction between the two selection treatments did not explain female reproductive investment (Table S3), further confirming the findings of the simpler model (see the main file, Results section).

Table S3: Effects of mating system and metapopulation structure evolutionary histories on reproductive tissue investment when including wet weight as a covariable. The table shows the output of Linear Mixed Models (LMMs) where polygamy is the reference level for the mating system treatment and the presence of population spatial structure is the reference level for the metapopulation structure treatment. p-values significant at <0.05 are in bold.

**Fixed Effects**	*β*	*Type II Wald* x^2	*Wald test df*	*P-value*
MALES				
Intercept	0.38			
Mating System	0.02	3.00	1	0.0831
Metapopulation Structure	0.02	2.04	1	0.1536
Wet Weight	0.06	58.12	1	**<0.001**
Mating System : Metapopulation Structure	-0.00	0.02	1	0.8750
Mating System : Wet Weight	-0.01	0.45	1	0.5032
Metapopulation Structure: Wet Weight	0.01	0.33	1	0.5629
FEMALES				
Intercept	0.59			
Mating System	0.07	10.22	1	**0.0014**
Metapopulation Structure	0.02	0.05	1	0.8209
Wet Weight	0.08	94.01	1	**<0.001**
Mating System: Metapopulation Structure	-0.04	1.21	1	0.2714
Mating System : Wet Weight	0.02	1.67	1	0.1959
Metapopulation Structure : Wet Weight	-0.03	3.46	1	0.0629

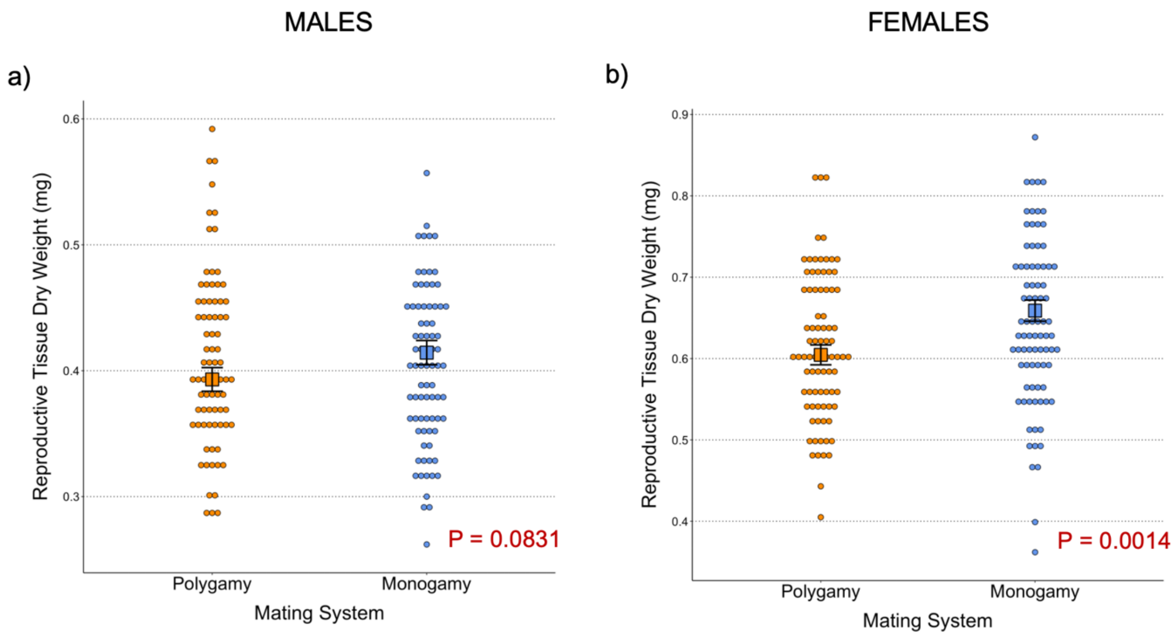

Figure S4: Marginal means from the models on (a) male and (b) female reproductive tissue investment with wet weight as a covariable. Each marginal mean is depicted as a square with its SE represented as error bars, whilst each group's distribution points are plotted around its mean. The p-values of the effect of mating system on reproductive tissue investment are displayed in red at the bottom right corner of each graph.

EVOLUTIONARY RESPONSES IN BODY WEIGHT

To test whether the selection regimes lead to divergence in body size, we ran LMMs with wet body weight as the response variable. As fixed effects we included the mating system treatment (two levels), the metapopulation structure treatment (two levels), and the interaction of these two factors. Line ID was included as a random effect with random intercepts.

Mating system was found to have a significant effect on male wet weight (χ^2 = 7.66, P = 0.0057; Cohen's *d* [95% CI] = 0.96 [-1.73, -0.19]; Table S4), with polygamous males being significantly heavier than monogamous males (Figure S5a). Although the effect of metapopulation structure was non-significant, it was marginally so (χ^2 = 3.09, P = 0.0786; Table S4). This effect was of medium size and only a relatively small tail of its confidence interval overlaps with zero (Cohen's *d* [95% CI] = 0.61 [-0.15, 1.37]), with males from metapopulation lines being heavier than those from undivided lines. Therefore, the possibility of metapopulation structure contributing to variation in male wet weight cannot

be fully ignored. Conversely, neither of the two selection treatments or their interaction were found to have a significant effect on female wet weight (Table S4). The direction of change attributable to mating system was the same as in males but non-significant (Figure S5b). The results from the body weight model further support our *a priori* decision to control for body weight in the analysis of reproductive tissue investment.

Table S4: Effects of mating system and metapopulation structure evolutionary histories on body size (i.e., wet body weight). The table shows the output of Linear Mixed Models (LMMs) where polygamy is the reference level for the mating system treatment and presence of population spatial structure is the reference level for the metapopulation structure treatment. p-values significant at <0.05 are in bold.

**Fixed Effects**	*β*	*Type II Wald* x^2	*Wald test df*	*P-value*
MALES				
Intercept	5.69			
Mating System	-0.56	7.66	1	**0.0057**
Metapopulation Structure	0.19	3.09	1	0.0786
Mating System : Metapopulation Structure	0.20	0.37	1	0.5413
FEMALES				
Intercept	7.53			
Mating System	-0.56	1.52	1	0.2183
Metapopulation Structure	0.14	1.23	1	0.2682
Mating System: Metapopulation Structure	0.38	0.42	1	0.5177

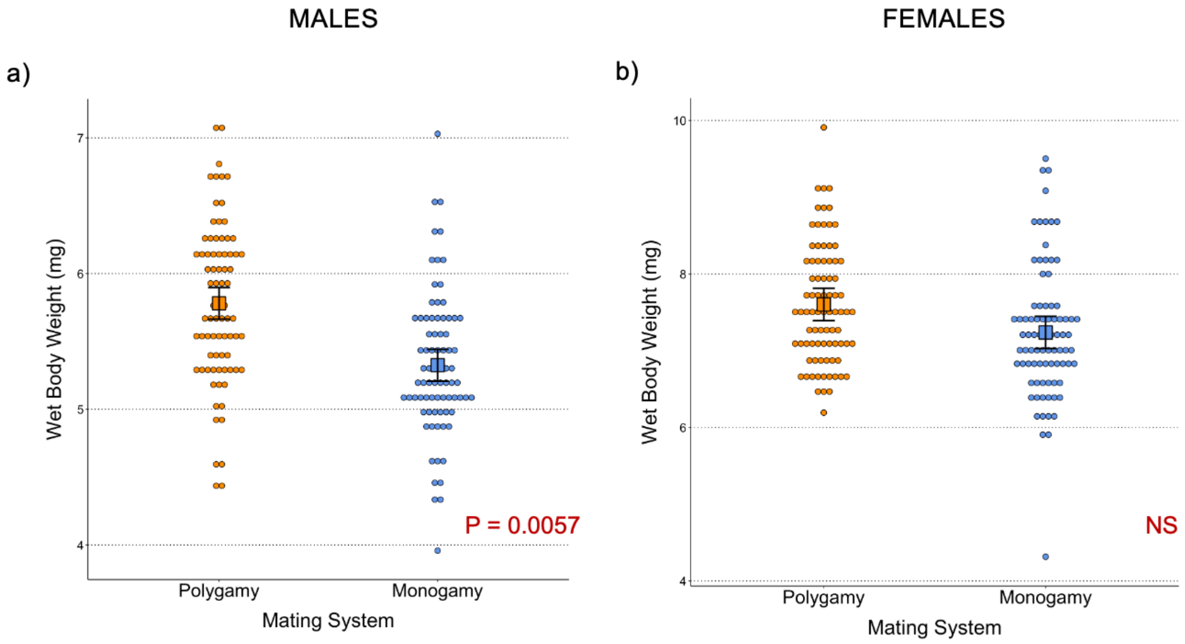

Figure S5: Marginal means from the models on (a) male and (b) female wet weight. Each marginal mean is depicted as a square with its SE represented as error bars, whilst each group's distribution points are plotted around its mean. The p-values of the effect of mating system on wet weight are displayed in red at the bottom right corner of each graph, with NS meaning no significance.

REPRODUCTIVE INVESTMENT VARIATION ACROSS TREATMENTS

To provide insight into the magnitude of reproductive investment in *C. maculatus*, we calculated dry reproductive tissue weight as a percentage of wet body weight according to sex. We found mean (± SE) reproductive tissue weight in *C. maculatus* to account for 7.32 ± 0.16% and 8.47 ± 0.23% of total wet body weight in males and females respectively. We additionally calculated the mean values for each treatment group and sex, as shown in Table S5.

Table S5: Dry reproductive tissue weight as a percentage of wet body weight depending on treatment and sex. Each value is calculated by grouping the data from all the individuals across the four selection lines for each of the sexual selection and metapopulation structure treatment combinations, with male and female means calculated separately. Each mean is displayed with its corresponding SE.

	**Metapopulation Structure Present**	**Metapopulation Structure Absent**
**Monogamy**	Males: 7.63 ± 0.21%<br>Females: 8.76 ± 0.26 %	Males: 7.50 ± 0.22%<br>Females: 8.94 ± 0.28%
**Polygamy**	Males: 7.26 ± 0.24%<br>Females: 8.18 ± 0.21%	Males: 6.89 ± 0.20%<br>Females: 8.00 ± 0.23%

References

1 Canal, D., Garcia-Gonzalez, F. & Garamszegi, L. Z. Experimentally constrained early reproduction shapes life history trajectories and behaviour. *Sci. Rep.* **11**, 4442 (2021). https://doi.org:10.1038/s41598-021-83703-1

2 Rodriguez-Exposito, E. & Garcia-Gonzalez, F. Metapopulation structure modulates sexual antagonism. *Evol lett* **5**, 344-358 (2021). https://doi.org:https://doi.org/10.1002/evl3.244

3 Zajitschek, S. R. K., Dowling, D. K., Head, M. L., Rodriguez-Exposito, E. & Garcia-Gonzalez, F. Transgenerational effects of maternal sexual interactions in seed beetles. *Heredity* **121**, 282-291 (2018). https://doi.org:10.1038/s41437-018-0093-y

4 Holland, B. & Rice, W. R. Experimental removal of sexual selection reverses intersexual antagonistic coevolution and removes a reproductive load. *Proc. Natl. Acad. Sci. U.S.A.* **96**, 5083-5088 (1999).

5 Martin, O. Y. & Hosken, D. J. Costs and benefits of evolving under experimentally enforced polyandry or monogamy. *Evolution* **57**, 2765-2772 (2003).

6 Becker, W. A. *Manual of quantitative genetics*. (Pullman, 1984).

7 Mohamed, M. I. *et al.* Ultrastructure and histopathological alteration in the ovaries of Callosobruchus maculatus (F.) (Coleoptera, Chrysomelidae) induced by the solar radiation. *The Journal of Basic & Applied Zoology* **68**, 19-32 (2015). https://doi.org:https://doi.org/10.1016/j.jobaz.2014.12.004

8 Oberauer, K. The Importance of Random Slopes in Mixed Models for Bayesian Hypothesis Testing. *Psychol Sci* **33**, 648-665 (2022). https://doi.org:10.1177/09567976211046884

9 Schielzeth, H. & Forstmeier, W. Conclusions beyond support: overconfident estimates in mixed models. *Behav. Ecol.* **20**, 416-420 (2009). https://doi.org:10.1093/beheco/arn145

10 Barr, D. J., Levy, R., Scheepers, C. & Tily, H. J. Random effects structure for confirmatory hypothesis testing: Keep it maximal. *J. Mem. Lang.* **68**, 255-278 (2013). https://doi.org:10.1016/j.jml.2012.11.001
